# Positive Allosteric Modulation of mGlu and Muscarinic Receptors Differentially Regulate Nitric Oxide-Dependent Signaling in a Scopolamine-Induced Model of Cognitive Impairment

**DOI:** 10.3390/medsci14060644

**Published:** 2026-10-07

**Authors:** Grzegorz Burnat, Michał Santocki, Anna Siekierzycka, Agata Płoska, Leszek Kalinowski, Joanna M. Wierońska

**Affiliations:** 1 Maj Institute of Pharmacology Polish Academy of Sciences, 12 Smetna Street, 31-343 Krakow, Poland; grzegorz.burnat@if-pan.edu.pl (G.B.); santocki@gmail.com (M.S.); 2 Department of Medical Laboratory Diagnostics—Fahrenheit Biobank BBMRI.pl, Medical University of Gdansk, 7 Debinki Street, 80-211 Gdansk, Poland; anna.siekierzycka@gumed.edu.pl (A.S.); agata.ploska@gumed.edu.pl (A.P.); 3 BioTechMed Center, Department of Mechanics of Materials and Structures, Gdansk University of Technology, 11/12 Narutowicza Steet, 80-223 Gdansk, Poland

**Keywords:** Alzheimer’s disease, scopolamine model, muscarinic receptors, mGlu receptors, nitric oxide (NO) signaling, cGMP pathway, positive allosteric modulators (PAMs)

## Abstract

**Background/Objectives**: The aim of the study was to evaluate the effects of positive allosteric modulators (PAMs) of metabotropic glutamate (mGlu2, mGlu5) and muscarinic (M1, M4) receptors on nitric oxide (NO֗)-dependent pathways. Scopolamine-induced cognitive impairment model reflecting cholinergic dysfunction characteristic of Alzheimer’s disease was used. **Methods**: The experiments were performed in vivo in Albino Swiss mice and in vitro in astrocytic cell lines (C8D1A, 1321N1). Chronic (14 days) scopolamine and mGlu2 (LY487379), mGlu5 (CDPPB), M1 (VU0357017), and M4 (VU0152100) PAMs were administered in vivo. In brain tissue samples (frontal cortex or hippocampus) cGMP levels (ELISA), total and protein-specific S-nitrosylation (biotin switch assay), and neuronal (nNOS) or endothelial (eNOS) synthase-dependent (NO֗) release (nanoelectrochemical detection) were measured. In vitro oxidative stress (NBT assay) was performed. **Results**: Significantly reduced cGMP levels, decreased both nNOS- and eNOS-dependent (NO֗) release, and lowered total protein S-nitrosylation in brain tissue were observed. mGlu2 and mGlu5 PAMs prevented the reduction in nNOS-related (NO֗) release and restored total S-nitrosylation. CDPPB did not protect against eNOS-related impairment. Muscarinic receptors modulators effectively counteracted eNOS dysfunction. All investigated compounds attenuated oxidative stress, with particularly pronounced effects in human astrocytes. **Conclusions**: mGlu and muscarinic receptor PAMs differentially modulate (NO֗)-related signaling pathways, demonstrating both therapeutic potential and potential safety limitations. None of the tested compounds fully restored neurochemical balance, suggesting that combination strategies (e.g., with (NO֗) donors or modulators of the (NO֗)-cGMP pathway) may represent a promising direction for future Alzheimer’s disease treatments.

## 1. Introduction

Alzheimer’s disease (AD) is the most destructive neurodegenerative disorder and represents a major and growing public health burden in aging societies. When the first clinical symptoms appear, substantial neuronal loss is already seen in cortical and hippocampal regions, which means that the treatment can only be symptomatic. Currently, there are no diagnostic methods that can detect the disease at an early stage, and the available therapy is chiefly based on symptom mitigation and slowing the progression of the disease. Currently approved anti-Alzheimer’s drugs include acetylcholinesterase inhibitors and NMDA receptor antagonists. In 2025, disease-modifying therapies (monoclonal antibodies) were approved that target the early stages of the disease by clearing toxic beta-amyloid plaques from the brain, thereby significantly slowing down cognitive decline. Despite this, novel therapeutic solutions are still needed.

Recent years have provided evidence that positive allosteric modulators of metabotropic glutamate (mGlu) and muscarinic (M) acetylcholine receptors may function as potential anti-Alzheimer’s agents [1,2,3,4,5,6,7,8,9,10,11]. These compounds may reverse scopolamine-induced memory impairments or improve memory in animal studies. However, behavioral data alone offers an incomplete picture of the drugs’ true action, overlooking the physiological and biochemical effects that ultimately dictate their long-term efficacy and safety. Crucially, pharmacological tools must be chosen not only to achieve a transient behavioral effect, but to directly target the underlying pathomechanisms of the disease. The pathophysiology of AD involves multiple mechanisms that complicate the treatment. Nitric oxide (NO֗)-dependent signaling is crucial in the process [12,13,14,15]. Physiologically, NO֗ acts as a critical signaling molecule involved in the regulation of neuronal communication, synaptic plasticity, and cerebrovascular function [16,17,18,19,20,21,22]. The molecule is synthesized after stimulation of constitutive NO֗ synthase isoforms—neuronal (nNOS) and endothelial (eNOS) after calcium (Ca^++^) influx, which activates NMDA receptors. This physiological pathway induces the generation of cyclic guanosine monophosphate (cGMP), a crucial intracellular second messenger that regulates a variety of processes. One of the most serious consequences of dysfunctional NO֗ neurotransmission is oxidative and nitrosative stress, which underlies the pathophysiology of the disorder [23,24,25,26] and develops when highly reactive superoxide (O_2_^−^•) is produced instead of NO֗. The simultaneous production of NO֗ and O_2_^−^• leads to the formation of peroxynitrite (ONOO^−^), a highly reactive oxidant triggering oxidative stress [23,24,25,26].

Another important NO֗-dependent mechanism is S-nitrosilation, the post-translational covalent attachment of an NO֗ group to cysteine thiol residues. When impairments in these S-nitrosylation processes occur, they lead directly to the dysregulation of key proteins central to Alzheimer’s pathology, such as glutamate transporter GLT-1 or βamyloid [27,28,29]. Concluding, the normalization of NO֗-signaling seems to be crucial in pharmacotherapy of AD.

Scopolamine, a well-established pharmacological tool for inducing AD-like cognitive deficits, has been shown to destabilize the eNOS pool, decrease L-glutamate and L-glutamine levels, and reduce L-arginine bioavailability—the primary substrate for NO֗ production—by promoting the synthesis of its methylated derivatives, including asymmetric dimethylarginine (ADMA), symmetric dimethylarginine (SDMA), and (NG)-monomethyl-L-arginine (NMMA). However, how scopolamine alters cGMP levels across specific brain structures and regulates S-nitrosylation processes—both critical in AD pathophysiology—remains unaddressed. To fill this gap, this study was undertaken to comprehensively evaluate the face and construct the validity of the scopolamine-induced model, and to investigate how potential anti-Alzheimer’s compounds, namely positive allosteric modulators (PAMs) of mGlu_2_, mGlu_5_, M_1_, and M_4_ receptors, modulate these scopolamine-induced alterations. Both receptor families are essential for synaptic plasticity, learning, and memory, and their functional interactions are closely linked to the glutamate– NO֗ –cGMP signaling axis. Given that these compounds have previously shown efficacy in animal models of cognitive decline, this selection allowed us to determine whether targeting distinct receptor subtypes differentially impacts NO֗ -dependent signaling under conditions of cholinergic dysfunction. Notably, the novelty of this study lies in demonstrating, for the first time, how these compounds modulate key parameters associated with the nitric oxide cascade, including cGMP levels, protein S-nitrosylation, oxidative stress, and, specifically, the release of nitric oxide dependent on nNOS or eNOS activity, following the administration of scopolamine alone or in combination with putative anti-AD compounds.

## 2. Materials and Methods

### 2.1. Animals

Male Swiss Albino mice, sourced from the Charles River Laboratory (Sulzfeld, Germany), served as the experimental subjects. The animals, weighing 20–25 g, were housed under controlled environmental parameters: a stable temperature of 22 ± 1 °C, a balanced 12 h light/dark regime, and ad libitum access to water and standard chow. The sample size for each experimental cohort was 8 individuals. All procedures strictly adhered to the guidelines outlined in EU Directive 2010/63/EU, as well as the national regulations established by the Polish Ministry of Agriculture and Rural Development.

### 2.2. Cell Lines

The C8-D1A mouse astrocytic cell line was obtained from LGC Standards, Łomianki, Poland (BSL: ATCC BSL 1. Lot/Batch Nr. 70043676) and 1321N1 human astrocytic cell line was obtained from Merck Life Sciences, Poznań, Poland (Nr: 86030402-1VL; 22I003/30025900).

### 2.3. Drugs

Compounds VU0357017 and VU0152100 were supplied by BioByrt (Cambridge, UK). Scopolamine and CDPPB were obtained from Abcam (Cambridge, UK), whereas LY487379 was sourced from Tocris (Bristol, UK). For administration, scopolamine, LY487379, and VU0357017 were prepared in 0.9% sterile saline. Conversely, CDPPB and VU0152100 were dissolved using a 10% aqueous solution of Tween 80. Vehicle control groups received equivalent injections of either 0.9% NaCl or 10% Tween 80 alone. All substances were delivered via intraperitoneal (i.p.) injection at a standard volume of 10 mL/kg. The selected working (top) doses, derived from prior protocols, were 1 mg/kg for scopolamine, 1 mg/kg for LY487379, 2 mg/kg for CDPPB, 0.5 mg/kg for VU0357017, and 0.25 mg/kg for VU0152100. Following a 14-day administration period, the frontal cortex (FC) and hippocampus were harvested for further analysis. The characterization of the compounds together with their behavioral activity is shown in Table 1. 

### 2.4. cGMP ELISA

Following sacrifice, brain tissues were promptly isolated, instantly frozen in liquid nitrogen, and kept at −80 °C prior to biochemical assays. To determine cGMP concentrations, tissue samples underwent homogenization in ice-cold 5% trichloroacetic acid (TCA) formulated in distilled water, ensuring both nucleotide stabilization and protein precipitation. The resulting homogenates were subjected to centrifugation at 3000× *g* for 5 min at 4 °C, after which the supernatants were harvested. Acetylated cyclic guanosine monophosphate (cGMP) content in the frontal cortex and hippocampus was measured using a competitive ELISA kit (Cayman Chemical, Ann Arbor, MI, USA; Cat. No. 581021) in strict accordance with the provider’s instructions. Standard calibration curves were established across a dynamic detection range of 0.023–3 pmol/1000 μL under the specific experimental parameters.

### 2.5. NBT Assay in Cell Cultures

Intracellular generation of reactive oxygen species (ROS) was evaluated utilizing the nitro blue tetrazolium (NBT) reduction assay. This technique is based on the reduction of NBT by superoxide anions, which yields insoluble, purple-blue formazan precipitates inside the cells. Post-incubation, these formazan crystals undergo solubilization and subsequent spectrophotometric quantification. Murine C8-D1A (ATCC) and human 1321N1 (ECACC) astrocytes were seeded into 96-well plates at an initial density of 4 × 10^4^ cells/well. The cultures were sustained in high-glucose DMEM (Gibco) enriched with 10% fetal bovine serum (BioWest, Nuaillé, France) and a penicillin–streptomycin cocktail (200 U/mL and 200 µg/mL, respectively; Merck). Following a 24 h attachment period, the cells were treated with the muscarinic receptor-positive allosteric modulators (VU0357017 or VU0152100), the mGlu_2_ receptor agonist LY487379, or the mGlu_5_ positive allosteric modulator CDPPB, each evaluated at three distinct concentrations (1, 5, and 10 µM). After 30 min of pre-incubation with the modulators, ROS production was induced by exposing the cells to scopolamine (50 µM) for 24 h. Phorbol 12-myristate 13-acetate (PMA; Merck Life Sciences, Poznań, Poland), a known protein kinase C activator, was employed as a positive control. At the end of the treatment period, an NBT chloride solution (10 mg/mL; Merck) was introduced into the wells, and the plates were kept at 37 °C for 1 h. The culture medium was then aspirated, and the cells were fixed with absolute methanol (15 min at room temperature) and air-dried. To dissolve the accumulated formazan deposits, 120 µL of 2 M KOH followed by 140 µL of DMSO was added to each well and mixed thoroughly. Finally, optical density was recorded at 620 nm using a microplate reader (Multiskan Spectrum, Thermo Fisher Scientific, Warsaw, Poland).

### 2.6. Detection of Protein S-Nitrosylation Using Biotin Switch Assay

To evaluate protein S-nitrosylation, a modified biotin switch approach was implemented, following the methodology established by Forrester. Prefrontal cortex specimens were homogenized in 1.8 mL of chilled lysis buffer (comprising 50 mM NaCl, 25 mM HEPES, 0.5 mM PMSF, 1% NP-40, and a protease inhibitor cocktail, pH 7.4). Homogenization was carried out using a TissueLyser II (Qiagen, Hilden, Germany) operating with 5 mm stainless steel beads at a frequency of 25 Hz for 3 min. The resulting lysates were cleared via a 10 min centrifugation at 2000× *g*, and total protein content was measured using the bicinchoninic acid (BCA) method. For the subsequent steps, an aliquot containing 1.5 mg of total protein per sample was utilized. Unreacted thiol groups were blocked by introducing 25% SDS and 20% methyl methanethiosulfonate (MMTS) into the homogenate diluted in HEN buffer, with the reaction proceeding at 50 °C for 20 min protected from light. Proteins were then precipitated with cold acetone and reconstituted in HENS buffer (100 mM HEPES, 1 mM EDTA, 0.1 mM neocuproine, 1% SDS, pH 8.0). Specific reduction of S-nitrosothiol bonds was performed by incubating the samples with 80 mM sodium ascorbate combined with biotin-HPDP (Sigma-Aldrich, Darmstadt, Germany) for 60 min at room temperature in the dark. After an additional acetone precipitation step, the biotin-tagged proteins were isolated by affinity purification using an avidin-conjugated resin overnight at 4 °C. Bound proteins were recovered by elution in HENS buffer at 95 °C for 10 min. Following a short spin (5000× *g*, 30 s), the supernatants were collected and mixed with 4× non-reducing Laemmli sample buffer. Biotinylated (S-nitrosylated) proteins were detected using Western blotting, employing a streptavidin-horseradish peroxidase conjugate (HRP; Abcam, Cambridge, UK; diluted 1:5000). The specificity and validation parameters of this assay are further detailed by Burnat et al. [30].

### 2.7. Western Blot Analysis

For total protein profiling, aliquots containing 10 µg of tissue lysate protein or 20 µL of the affinity-purified biotinylated fraction were resolved via 8% sodium dodecyl sulfate–polyacrylamide gel electrophoresis (SDS-PAGE) and electroblotted onto nitrocellulose membranes. Non-specific binding sites were blocked for 1 h at room temperature using TBS-T supplemented with 5% bovine serum albumin (BSA, Fraction V). Subsequently, the membranes were incubated with primary antibodies for 2 h at room temperature. The primary antibody panel comprised anti-β-actin (Sigma-Aldrich; 1:10,000), anti-amyloid precursor protein (APP; Millipore, Poznań, Poland; 1:2000), anti-glutamate transporter 1 (GLT-1; Millipore; 1:3000), and anti-tau (Abcam, Gdańsk, Poland; 1:2000). Following a series of washes, the membranes were exposed to appropriate HRP-conjugated secondary antibodies (Promega, Warsaw, Poland 1:5000; Millipore, 1:10,000) for 1 h at room temperature. Protein bands were visualized using the SuperSignal™ West Pico PLUS chemiluminescent substrate (Thermo Scientific, Schwerte, Germany) and captured on a GeneGnome XRQ imaging system (Syngene, Cambridge, UK). Densitometry of the immunoreactive signals was carried out using GnomeSys software (v1.8.2). Representative Western blot images are provided in the Supplementary Materials.

The samples were prepared and processed in independent experimental batches, which may have contributed to some variability in β-actin signal intensity between blots. Importantly, protein concentration was determined using the BCA assay, and an equal amount of total protein (10 µg per sample) was loaded onto the SDS-PAGE gels. In addition, total protein staining with amido black was performed and is provided in the original blot documentation as an independent assessment of protein loading.

In the original analysis, the investigated proteins were normalized to β-actin. We acknowledge the observed variability of β-actin and have provided the original Western blot images, including the corresponding total protein staining, for evaluation.

### 2.8. Measurement of NO֗ Release

Electrochemical quantification of •NO release was performed using a porphyrin nanosensor integrated into a standard three-electrode configuration, which comprised a working electrode, an Ag/AgCl reference electrode, and a platinum counter electrode. The system was coupled with a Gamry VFP600 multichannel potentiostat (Gamry Instruments, Warminster, PA, USA). Prior to the electrochemical assays, tissue specimens were thawed and equilibrated for 30 min at 37 °C in HBSS buffer supplemented with 5% FBS and 100 µM L-arginine. A micromanipulator was employed to position the tip of the NO nanosensor adjacent to the tissue surface (at a distance of 5 ± 2 µm). Variations in the differential pulse voltammetry current were recorded at 0.65 V, the characteristic peak potential corresponding to NO oxidation, following stimulation with 1 µmol/L calcium ionophore (A23187). To assess enzyme specificity, measurements were repeated after an additional 30 min incubation at 37 °C in the presence of selective inhibitors: either 3 µM NPA (Nω-propyl-L-arginine; a specific nNOS inhibitor) alone, or a combination of 3 µM NPA and 300 µM L-NAME (Nω-nitro-L-arginine methyl ester; a non-specific NOS inhibitor).

## 3. Results

### 3.1. cGMP Level

**Figure 1:**
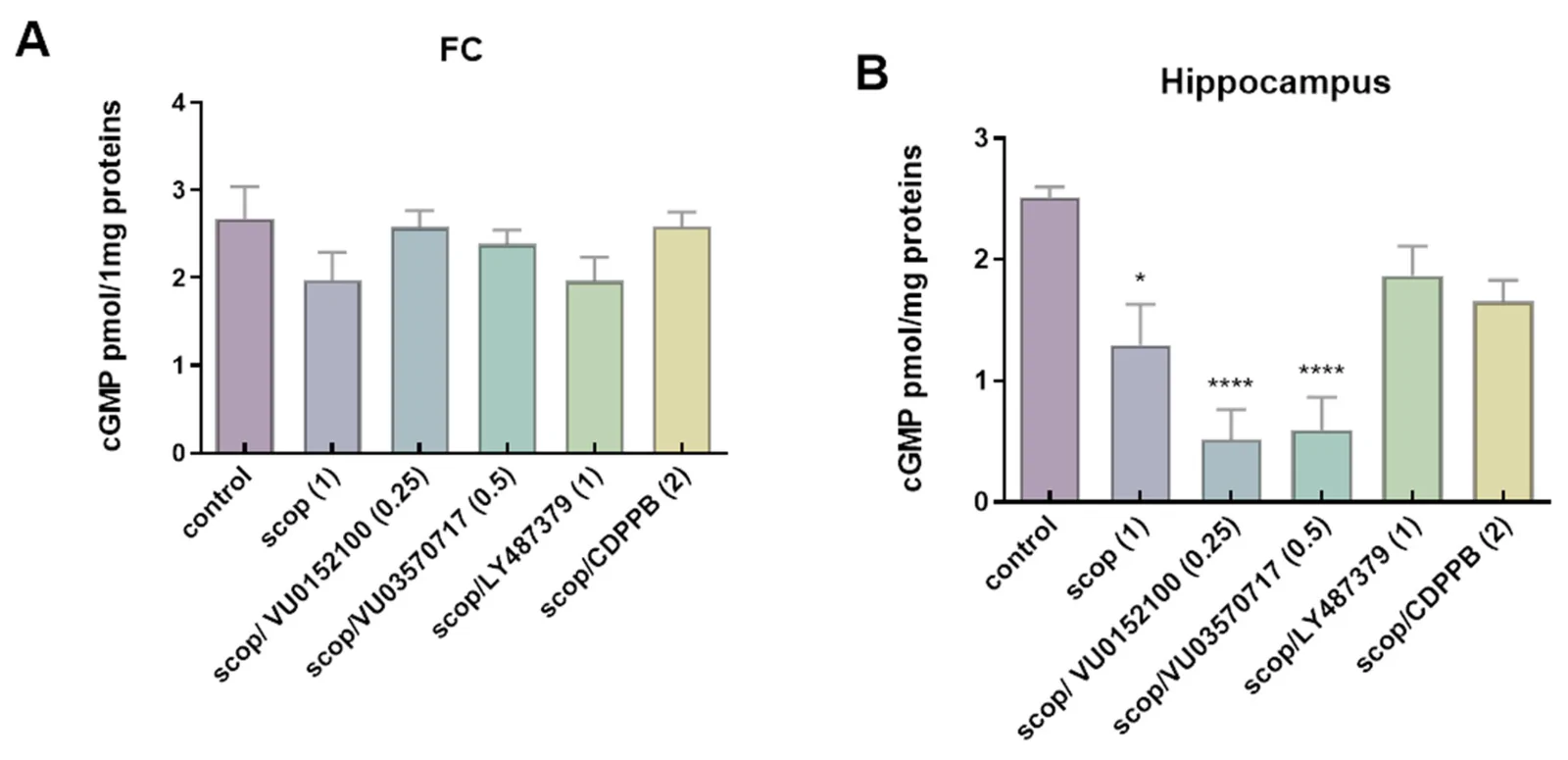
cGMP level in the frontal cortex (**A**) and hippocampus (**B**) after chronic administration (14-day) of scopolamine alone or in combination with the investigated compounds. Treatments with doses (in brackets, mg/kg) are indicated below the bars. Data are presented as mean ± SEM. * *p* < 0.01; **** *p* < 0.0001 vs. control.

As revealed by one-way ANOVA followed by Dunnett’s multiple comparisons test, none of the treatments significantly affected cGMP levels in the FC compared with the control group (F_(5.42)_ = 1.52, *p* = 0.200). In the hippocampus, a significant decrease in cGMP levels was observed following administration of scopolamine alone, as well as scopolamine in combination with VU0357017 or VU0152100 (F_(5.41)_ = 10.34, *p* < 0.0001). Neither LY487379 nor CDPPB reversed this deficit, as the effect of the compounds was not statistically significant F_(2.20)_ = 1.21; *p* = 0.31. The data are presented in Figure 1.

Overall: No treatments significantly changed cGMP levels in the frontal cortex, scopolamine—both alone and combined with VU0357017 or VU0152100—significantly decreased cGMP levels in the hippocampus. This deficiency was not reversed by LY487379 or CDPPB.

### 3.2. NBT Assay

In the mouse astrocytic cell line (C8D1A), exposure to scopolamine (50 µM) induced an oxidative burst comparable to that produced by the reference compound PMA (200 µM). Among the tested compounds, only VU0357017 prevented this effect as determined by one-way ANOVA followed by Tukey’s post hoc test (F_(6.53)_ = 18.78, *p* < 0.0001).

**Figure 2:**
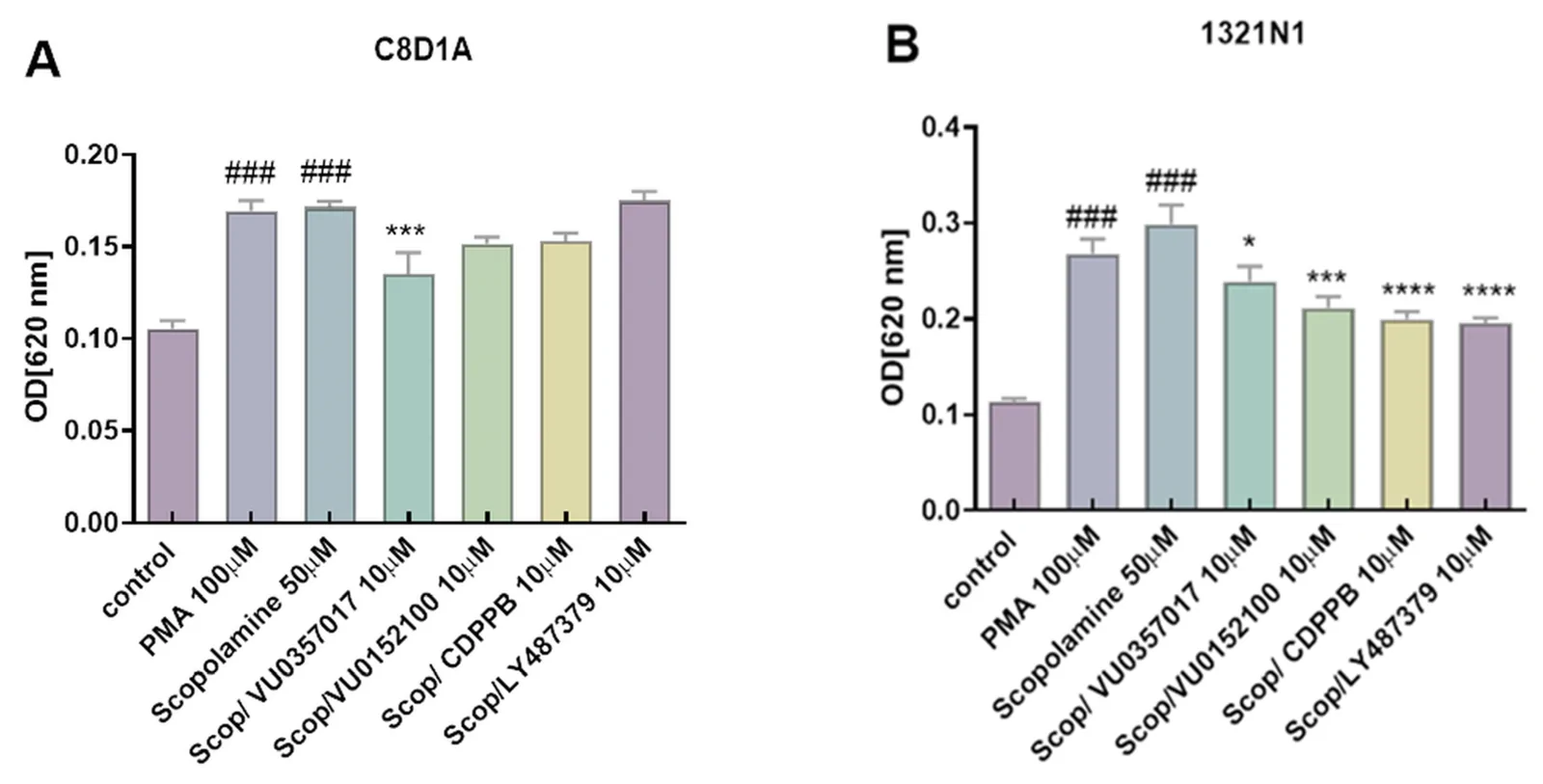
NBT assay results in murine (**A**) and human (**B**) astrocytic cell lines following exposure to scopolamine alone or in combination with the investigated compounds, indicated below the bars. Data are presented as mean ± SEM. Symbols denote statistical significance: ### *p* < 0.0001 vs. control; * *p* < 0.02, *** *p* < 0.003, **** *p* < 0.0001 vs. scopolamine.

Similarly, exposure to scopolamine (50 µM) in a 1321N1—human astrocytic cell line also induced a comparable oxidative burst. However, contrary to the C8D1A cell line, all investigated compounds significantly prevented this effect at concentrations of 1–10 µM, as revealed by one-way ANOVA analysis (F_(6.50)_ = 23.87, *p* < 0.0001). The tested compounds showed the same activity at all verified concentrations. As there is no need to present three identical bars, we present the data and statistical analysis only for the highest concentration of 10 µM. These data are presented in Figure 2.

Overall: While scopolamine induced a comparable oxidative burst in both astrocytic cell lines, only VU0357017 prevented this effect in the mouse line (C8D1A), whereas all tested compounds (10 µM) significantly blocked it in the human line (1321N1).

### 3.3. S-Nitrosylation in Brain Tissue Samples

Prior to evaluating S-nitrosylation of individual proteins, samples were separated by SDS-PAGE, and total S-nitrosylation levels were compared across treatment groups. No significant differences were observed between groups.

Total levels of analyzed proteins within six treatment groups were estimated as relative values, normalized to β-actin levels. Chronic scopolamine administration did not alter the expression of glutamate transporter 1 (GLT-1) compared with the control, nor did co-administration with VU0152100 or VU0357017. In contrast, as determined by one-way ANOVA followed by Tukey’s post hoc comparison, co-treatment with LY487379 and CDPPB significantly decreased GLT-1 expression, (F_(5.42)_ = 13.12, *p* < 0.0001).

Decreased expression of amyloid precursor protein (APP) was observed following scopolamine treatment. Only VU0357017 prevented this decrease, as determined by one-way ANOVA followed by Tukey’s post hoc test (F_(5.43)_ = 11.18, *p* < 0.0001); the other compounds had no significant effect.

Total GLT1-SNO levels were increased following scopolamine administration, and co-treatment with VU0152100, LY487379, or CDPPB prevented this effect (F_(5.43)_ = 21.64, *p* < 0.0001).

**Figure 3:**
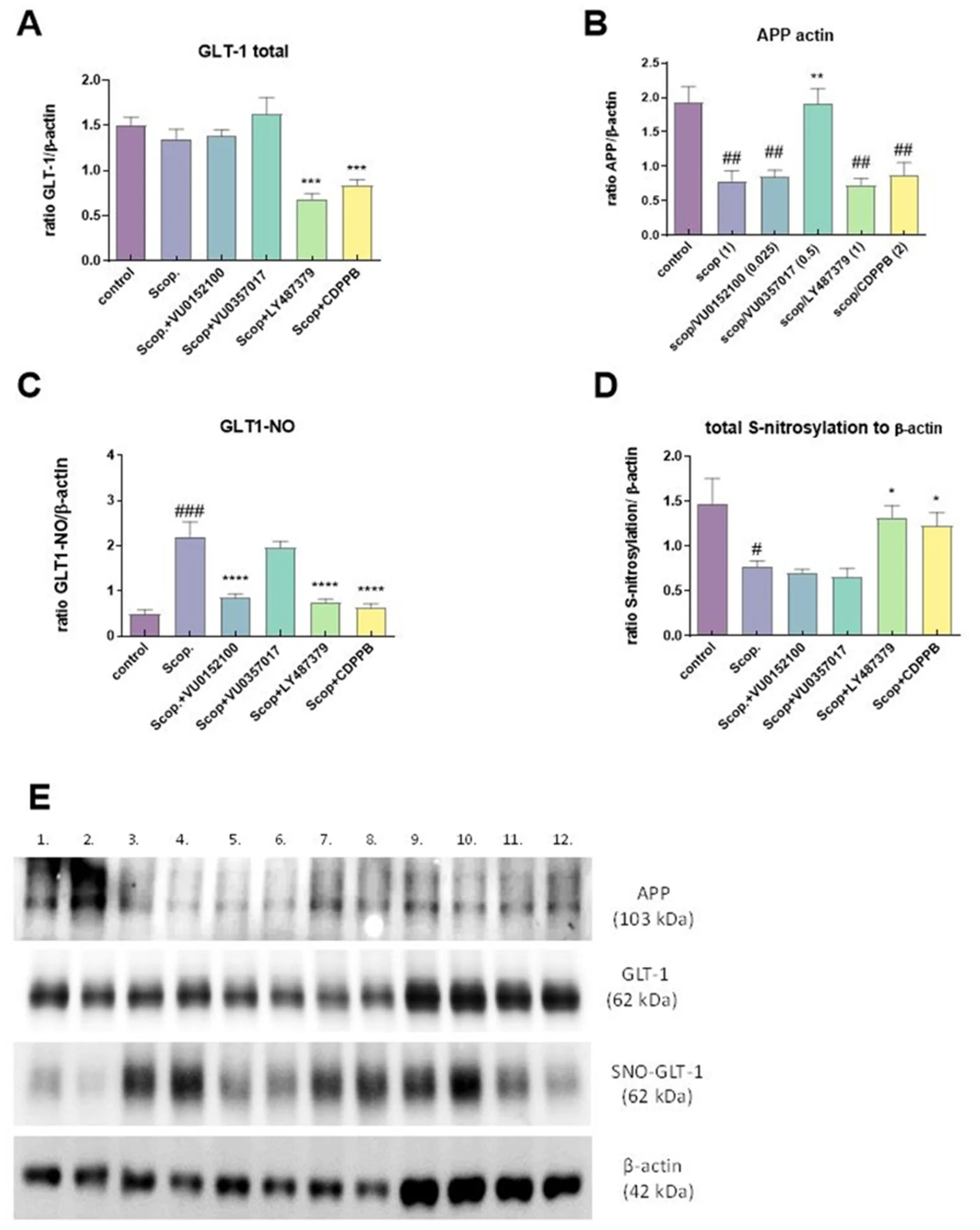
Densitometric analysis of Western blot results: (**A**)—total glutamate transporter 1 protein (GLT-1); (**B**)—amyloid precursor protein (APP); (**C**)—GLT1-SNO; (**D**)—total S-nitrosylation in the frontal cortex samples after chronic administration (14-day) of the investigated compounds; (**E**)—representative blots (lines: 1, 2—control; 3, 4—scopolamine; 5, 6—Scop.+VU0152100; 7, 8—Scop.+VU0357017; 9, 10—Scop.+LY487379; 11, 12—Scop.+CDPPB). Data are presented as mean ± SEM. Symbols indicate statistical significance: # *p* < 0.01, ## *p* < 0.001 and ### *p* < 0.0001 vs. control; * *p* < 0.05, ** *p* < 0.01, *** *p* < 0.001 and **** *p* < 0.0001 vs. scopolamine.

Total S-nitrosylation levels were decreased following scopolamine treatment; this decrease was prevented by LY487379 and CDPPB, but not by muscarinic receptor-positive modulators (F_(5.43)_ = 5.1, *p* < 0.0009). These data are presented in Figure 3.

Overall: While total protein S-nitrosylation remained unchanged across groups, scopolamine treatment decreased APP expression and increased GLT-1 S-nitrosylation. These alterations were selectively prevented by specific compound treatments, whereas combined LY487379 and CDPPB administration significantly reduced total GLT-1 expression.

### 3.4. Electrochemical Detection of NO֗ Release

**Figure 4:**
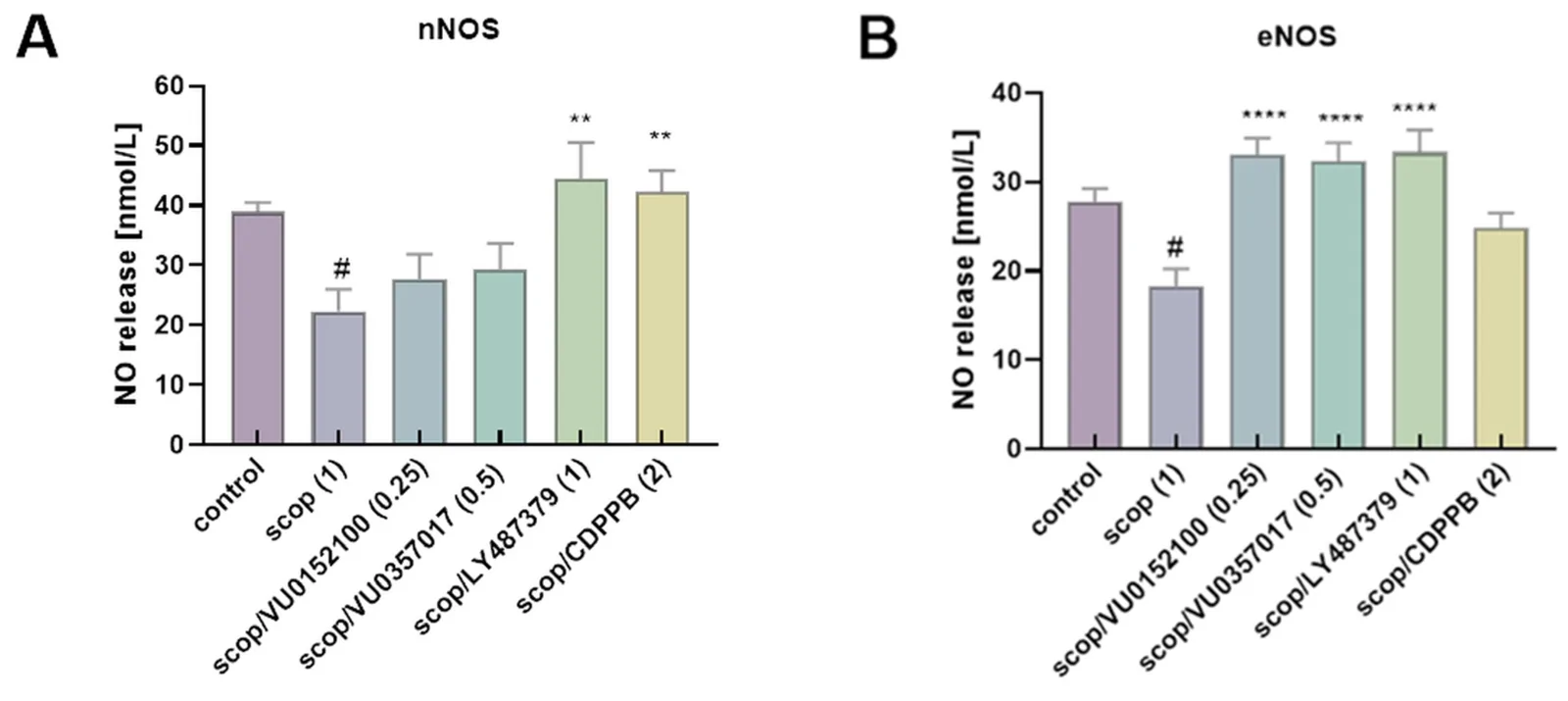
Electrochemical detection of NO֗ release in brain tissue. (**A**) nNOS-dependent NO֗ release in brain tissue from control mice and mice treated with scopolamine (1 mg/kg) alone or in combination with VU0152100 (0.25 mg/kg), VU0357017 (0.5 mg/kg), LY487379 (1 mg/kg) or CDPPB (2 mg/kg). NO release was measured following stimulation with 1 µmol/L calcium ionophore (CaI) twice, before and after pre-incubation with 3 µM NPA. (**B**) eNOS-dependent NO release in brain tissue from control mice and mice treated with scopolamine (1 mg/kg) alone or in combination with VU0152100 (0.25 mg/kg), VU0357017 (0.5 mg/kg), LY487379 (1 mg/kg) or CDPPB (2 mg/kg). NO release was measured following stimulation with 1 µmol/L CaI twice: after pre-incubation with 3 µM NPA and with 3 µM NPA and 300 µM L-NAME. Data are expressed as mean ± SEM of three independent measurements. Symbols indicate statistical significance: # *p* < 0.002 vs. control; ** *p* < 0.007, **** *p* < 0.0001 vs. scopolamine.

Scopolamine significantly decreased nNOS-dependent NO֗ release (Figure 4A). Neither M1 nor M4 PAM reversed this effect. Co-treatment with mGlu_2_ or mGlu_5_ ligands significantly prevented the scopolamine-induced reduction, as determined via one-way ANOVA followed by Dunnett’s multiple comparisons test (F_(5.42)_ = 4.52, *p* < 0.002).

Scopolamine significantly decreased eNOS-dependent NO֗ release, and this effect was prevented by co-treatment with VU0357017, VU0152100 or LY487379 (Figure 4B). The effect of CDPPB did not reach statistical significance (F_(5.42)_ = 9.33, *p* < 0.0001).

Overall: Scopolamine significantly decreased both nNOS- and eNOS-dependent NO release, with the nNOS deficit prevented by mGlu2 or mGlu5 ligands and the eNOS deficit blocked by VU0357017, VU0152100, and LY487379.

## 4. Discussion

**Validity of the Scopolamine Model and the Activity of mGlu or Muscarinic Ligands**

Scopolamine serves as a premier pharmacological model of Alzheimer’s disease (AD) by temporarily blocking muscarinic acetylcholine receptors, thereby directly mimicking the cholinergic dysfunction that precipitates cognitive decline in AD patients [31]. This non-genetic, reversible approach rapidly induces dementia-related cognitive and biochemical deficits in both human volunteers and animal models [32,33,34,35]. Prior research has demonstrated that scopolamine accelerates the production of L-arginine derivatives, enhances oxidative stress, and elevates amyloid-beta (Aβ) levels—all established hallmarks of AD (Table 2).

The findings of the present study further extend this characterization at the molecular level. Specifically, we demonstrate that chronic scopolamine administration decreases cyclic guanosine monophosphate (cGMP) production in key cognitive brain structures, alters the S-nitrosylation of proteins crucial to AD pathogenesis, and inhibits both neuronal (nNOS) and endothelial (eNOS) nitric oxide synthase-related NO֗ release. Although behavioral testing was not explicitly performed in this study, the selected scopolamine regimen (1 mg/kg for 14 days) has been thoroughly linked to cognitive deficits in our previous work, in which the investigated compounds successfully counteracted these impairments (Table 1) [3,4,5]. Together, these robust biochemical shifts confirm the high face and construct validity of this pharmacological model, supporting its utility in the search for novel anti-AD therapeutics.

**Cholinergic–Glutamatergic Cross-Talk and Synaptic Plasticity**

Beyond primary cholinergic deficits, a progressive malfunction of glutamatergic neurotransmission heavily contributes to cognitive decline in AD [36,37,38]. Emerging evidence highlights that reciprocal interactions between acetylcholine and glutamate are crucial for learning and memory processes; acetylcholine facilitates glutamatergic activity by coordinating the acquisition and recall phases within the cortex and hippocampus. Central to these memory processes is long-term potentiation (LTP), a cellular mechanism of synaptic plasticity strictly regulated by the glutamate– NO֗ –cGMP signaling axis.

Scopolamine is known to disrupt LTP [39,40,41,42] as well as the discrete molecular components that drive this process. Previously, no changes in nNOS expression as measured by monomer/dimers ratio was reported [43]. As demonstrated here, it reduces cGMP levels and nNOS-dependent NO֗ release, thereby complementing the decreased L-glutamate levels reported in our previous studies (Table 2). Given that both cholinergic and glutamatergic receptors tightly regulate LTP [44,45,46,47], PAMs capable of fine-tuning these systems represent a viable strategy to restore signaling homeostasis. The PAMs investigated in this study—targeting M_1_ (VU0357017), M_4_ (VU015100), mGlu_2_ (LY487379), and mGlu_5_ (CDPPB) receptors—or their structural analogs, could therefore be considered promising candidates for anti-AD drug development. While their ability to prevent scopolamine-induced memory dysfunctions in animal models has been established (Table 1) [3,4,5], further data are required to elucidate how these specific ligands modulate the scopolamine-induced disruption of LTP.

**Efficacy of PAMs on Neurochemical Deficits and eNOS Regulation**

Our previous findings demonstrated that chronic scopolamine administration induces eNOS uncoupling in both the cortex and hippocampus—an effect prevented by M_1_ or M_4_ receptor ligands. In contrast, the mGlu_2_ PAM exhibited minimal efficacy, whereas the mGlu_5_ PAM, CDPPB, further exacerbated the detrimental effects of scopolamine. Consistent with those observations, the present study revealed a distinct scopolamine-induced reduction in eNOS-mediated NO֗ release within the murine cortex. Administration of M_1_, M_4_, or mGlu_2_ PAMs effectively reversed this endothelial deficit, whereas CDPPB exerted no discernible effect, closely aligning with our prior reports [3,4,5].

At the cellular level, we report that the investigated ligands effectively attenuate scopolamine-induced oxidative stress in human astrocytic cell lines. The differential responses observed between C8D1A and 1321N1 astrocytes to the investigated PAMs may reflect underlying differences in species origin, receptor expression profiles, receptor–signaling coupling efficiency, basal redox states, or other cell-line-specific characteristics. Although these individual parameters were not directly examined in the present study and require further mechanistic elucidation, these findings provide valuable insights into the intrinsic antioxidative capacities of these compounds. Notably, none of the evaluated compounds rescued the scopolamine-mediated decline in cGMP production—a deficit known to directly accelerate downstream oxidative stress cascades. Intriguingly, ligands targeting the M_1_ and M_4_ receptors further aggravated this specific scopolamine-induced impairment.

This profound imbalance between cGMP production and oxidative stress carries severe consequences for brain neurochemistry, particularly regarding alterations in S-nitrosylation processes. S-nitrosylation modulates protein function and intracellular signaling by dynamically altering protein conformation, subcellular localization, enzymatic activity, and protein–protein interactions. Investigating these pathways poses significant technical challenges due to the highly transient and labile nature of S-nitrosocysteine modifications. In this study, scopolamine administration decreased both total S-nitrosylation and the specific S-nitrosylation of amyloid precursor protein (APP) in brain homogenates, while concurrently elevating GLT-1 S-nitrosylation (GLT-1-SNO). Aberrant S-nitrosylation of these target proteins may trigger localized neuroinflammatory responses and disrupt glutamatergic transmission, both of which are established features of AD progression [48,49,50]. The investigated compounds exerted highly divergent effects on these molecular parameters, thereby precluding a definitive conclusion regarding which specific agent most effectively normalizes scopolamine-induced dysregulation of S-nitrosylation pathways.

**The Role of Muscarinic or mGlu Receptors PAMs in the Vicious Circle of AD Pathogenesis**

Overall, positive allosteric modulation of muscarinic or mGlu receptors has the potential to normalize the complex pathophysiology of AD, which involves multiple NO֗-related impairments. Beyond well-established perturbations within the acetylcholinergic and glutamatergic networks, eNOS dysfunction represents a pivotal driver in AD pathogenesis. Under physiological conditions, eNOS functions strictly as an active homodimer, where eNOS-derived NO֗ maintains essential neurovascular coupling and modulates neurotransmitter release. However, pathological uncoupling—precipitated by L-arginine or cofactor depletion alongside Aβ accumulation—shifts enzymatic activity away from NO֗ production and toward the aberrant generation of superoxide anions.

**Figure 5:**
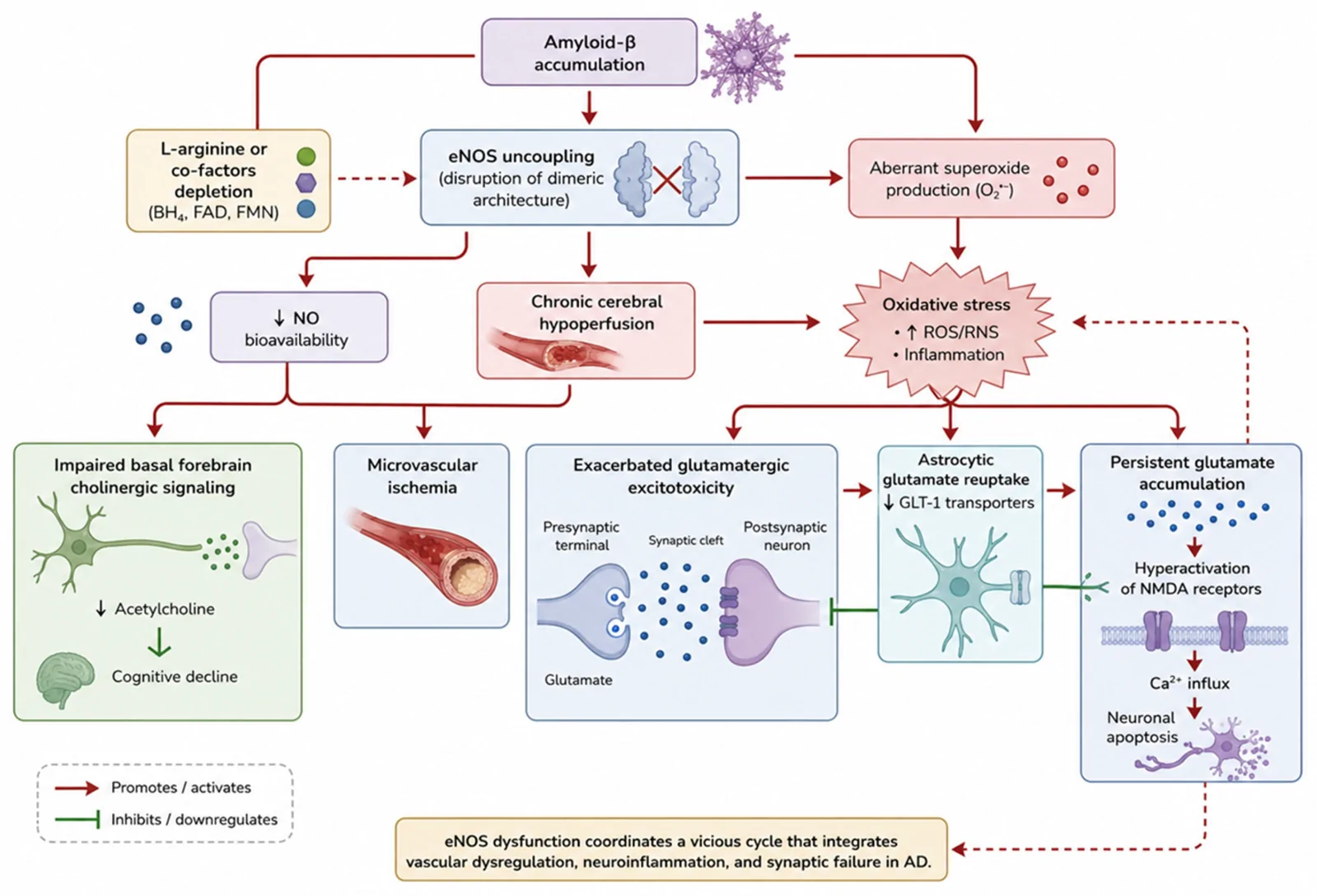
Schematic of the cascade of events establishing a vicious cycle that contributes to the pathophysiology of Alzheimer’s disease, as described in the discussion section.

This phenomenon of eNOS uncoupling severely disrupts the dimeric architecture of the enzyme. The resulting elevated oxidative stress induces chronic cerebral hypoperfusion and exacerbates localized oxidative damage, which synergistically impairs basal forebrain cholinergic signaling, thereby accelerating acetylcholine deficits and cognitive decline. Furthermore, this endothelial impairment exacerbates glutamatergic excitotoxicity; the attendant microvascular ischemia compromises astrocytic glutamate reuptake via the downregulation of GLT-1 transporters. This leads to persistent synaptic glutamate accumulation, hyperactivation of NMDA receptors, and subsequent calcium-dependent neuronal apoptosis. Consequently, eNOS dysfunction does not merely coexist with neurotransmitter deficits, but actively orchestrates a destructive cascade linking vascular dysregulation, neuroinflammation, and synaptic failure in AD. Among the investigated compounds, the mGlu_2_ PAM exerted the best activity, and mGlu_5_ was the most questionable. Muscarinic ligands were not effective at all important points. A schematic integration of these interconnected pathways is illustrated in Figure 5, and the exact activity of ligands is described in Table 2.

## 5. Conclusions

In conclusion, this study confirms that chronic scopolamine administration serves as a highly valid pharmacological model of AD by accurately replicating core pathological features, including oxidative stress, impaired cGMP production, and decreased eNOS and nNOS-related NO֗ release. These results are in line with expectations. The ability of the muscarinic or mGlu PAMs to prevent scopolamine-induced decrease in NO֗ release and the modification of S-nitrosilation processes in murine brains have been demonstrated, as has their ability to prevent scopolamine-induced oxidative stress in vitro. These results are in line with expectations and show their potency to normalize NO֗-related biochemical parameters. However, the potency of mGlu ligands was much better and more promising. Conversely, the activity of the compounds in relation to cGMP changes was much more disappointing. Taking into consideration our previous results, future translational drug designs must be carefully considered and each compound, with its pros and cons, must be balanced to achieve maximal beneficial therapeutic effects against potentially unfavorable biochemical outcomes in the treatment of AD. One solution is the use of combination strategies, including co-administration with NO֗ donors. These represent a subsequent phase of investigation that has been partially explored in our previous work and is explicitly highlighted in the discussion as a prominent direction for future research. We have shown, both here and in previous research, that top doses may have a minimal effect on biochemical changes linked with AD pathophysiology.

## Figures and Tables

**Table 1:** Pharmacological characteristics and behavioral activity of investigated compounds. NOR-novel object recognition.

Compound	Properties	Behavioral Activity After Acute Injection (NOR)	Behavioral Activity After Chronic (14 Days) Injection (NOR)	Refs.
**Scopolamine hydrobromide** *(α,S)-α-(Hydroxymethyl) benzeneacetic acid (1α,2β,4β,5α,7β)-9-methyl-3-oxa-9-azatricyclo [3.3.1.02,4]non-7-yl ester*	Nonselective muscarinic antagonist	Induced cognitive dysfunction at the dose 1 mg/kg	Induced cognitive dysfunction at the dose 1 mg/kg	[3,4,5]
**VU0357017** *ethyl 4-[[2-[(2-methylbenzoyl) amino] ethyl] amino]–1-piperidinecarboxylate*	Selective allosteric agonist of M_1_ muscarinic receptor with EC_50_ = 477 nM	Reversed scopolamine-induced cognitive decline at the dose 0.1 and 0.5 mg/kg	Reversed scopolamine-induced cognitive decline at the dose 0.05 and 0.1 mg/kg	[5]
**VU0152100** *3-amino-N-(4-methoxybenzyl)-4,6-dimethylthieno[2,3-b]pyridine-2-carboxamide*	Selective positive allosteric agonist of M_4_ muscarinic receptor with EC_50_ = 380 nM	Reversed scopolamine-induced cognitive decline at the dose 0.1 and 0.25 mg/kg	Reversed scopolamine-induced cognitive decline at the dose 0.025 and 0.1 mg/kg	[5]
**CDPPB** *3-Cyano-N-(1,3-diphenyl-1H-pyrazol-5-ylbenzamide*	mGlu_5_ positive allosteric modulator; EC_50_ values are 10 and 20 nM for human and rat, respectively	Reversed scopolamine-induced cognitive decline at the dose 1 and 2 mg/kg	Reversed scopolamine-induced cognitive decline at the dose 0.5 and 2 mg/kg	[3,4]
**LY487379** *N-(4-(2-Methoxyphenoxy)phenyl)-N-(2,2,2-trifluoroethylsulfonyl) pyrid-3-ylmethylamine hydrochloride*	mGlu_2_ positive allosteric modulator; EC_50_ = 1.7 μM	Reversed scopolamine-induced cognitive decline at the dose 0.5 and 1 mg/kg	Reversed scopolamine-induced cognitive decline at the dose 0.1 and 1 mg/kg	[3,4]

**Table 2:** Schematic summary of the effects of scopolamine and muscarinic or mGlu receptor ligands on nitric oxide-regulated parameters after chronic (14 days) administration, at the doses indicated as mg/kg in parentheses. Data were compiled from the present study and previous works [3,4,5]. Arrows indicate the direction of the observed change, whereas “normalization” indicates a statistically significant reversal toward control values. The schematic is intended as an overview of the main findings and should not be interpreted as a quantitative comparison between treatments or as indicating uniform effects across brain regions, doses, or experimental conditions. VU0357017-M_1_ allosteric agonist; VU0152100-M_4_ PAM; LY487379-mGlu_2_ PAM; CDPPB-mGlu_5_ PAM. ↓ decrease; ↑ increase; ↔ normalization. n.s—the result was not statistically significant; n.a—results are not available.

		Scopolamine (1)		Scopolamine/ VU0357017 (0.5)		Scopolamine/ VU0152100 (0.25)		Scopolamine/ LY487379 (1)		Scopolamine/ CDPPB (2)	
		Cx	hipp	Cx	hipp	Cx	hipp	Cx	hipp	Cx	hipp
**Oxidative stress (in vitro)**		**↑**		**↔**		**↔**		**↔**		**↔**	
**cGMP**		**n.s**	**↓**	**↔**	**↓**	**↔**	**↓**	**↔**	**↓**	**↔**	**↓**
**NO release**	**eNOS**	**↓**	n.a	**↔**	n.a	**↔**	n.a	**↔**	n.a	**↓**	n.a
	**nNOS**	**↓**	n.a	**↓**	n.a	**↓**	n.a	**↔**	n.a	**↔**	n.a
**s-nitrosilation**	**total**	**↓**	n.a	**↓**	n.a	**↓**	n.a	**↔**	n.a	**↔**	n.a
	**APP**	**↓**	n.a	**↔**	n.a	**↓**	n.a	**↓**	n.a	**↓**	n.a
	**GLT-1**	**↑**	n.a	**↓**	n.a	**↔**	n.a	**↔**	n.a	**↔**	n.a
**L-arginine derivatives**	**ADMA**	**↓**	n.s	**↓**	**↓**	**↓**	↔	**↓**	**↓**	**↓**	**↓**
	**SDMA**	**↓**	**↓**	**↓**	**↔**	**↓**	**↓**	**↓**	**↓**	**↓**	**↓**
	**NMMA**	**↓**	**↓**	**↓**	**↔**	**↔**	**↔**	**↓**	**↓**	**↓**	**↓**
**L-aminoacids**	**L-glutamate**	**↓**	**↓**	**↓**	**↓**	**↓**	**↓**	**↓**	**↓**	**↓**	**↓**
	**L-glutamine**	**↓**	**↓**	**↓**	**↓**	**↓**	**↓**	**↓**	**↓**	**↓**	**↓**
**eNOS monomers uncoupling**		**↑**	n.s	**↔**	**↓**	**↔**	**↓**	**↔**	**↔**	**↑**	n.s

## Data Availability

The data generated and analyzed during the current study are available from the corresponding authors on reasonable request.

## Supplementary Materials

The following supporting information can be downloaded at https://www.mdpi.com/article/10.3390/medsci14060644/s1, Original Western blot images corresponding to the data presented in Figure 3.

## Abbreviations

The following abbreviations are used in this manuscript:

A23187	Calcium ionophore (calcimycin)
AD	Alzheimer’s disease
ADMA	Asymmetric dimethylarginine
APP	Amyloid precursor protein
BCA	Bicinchoninic acid
BSA	Bovine serum albumin
CaI	Calcium ionophore
CDPPB	mGlu5 receptor positive allosteric modulator
cGMP	Cyclic guanosine monophosphate
DMEM	Dulbecco’s Modified Eagle Medium
DMSO	Dimethyl sulfoxide
EDTA	Ethylenediaminetetraacetic acid
ELISA	Enzyme-linked immunosorbent assay
eNOS	Endothelial nitric oxide synthase
FBS	Fetal bovine serum
FC	Frontal cortex
GLT-1	Glutamate transporter 1
HBSS	Hank’s balanced salt solution
HEN	HEPES–EDTA–neocuproine buffer
HENS	HEPES–EDTA–neocuproine–SDS buffer
HEPES	4-(2-Hydroxyethyl)-1-piperazineethanesulfonic acid
HRP	Horseradish peroxidase
L-NAME	Nω-Nitro-L-arginine methyl ester
LTP	Long-term potentiation
LY487379	mGlu2 receptor positive allosteric modulator
M1	Muscarinic acetylcholine receptor M1
M4	Muscarinic acetylcholine receptor M4
mGlu	Metabotropic glutamate
mGlu2	Metabotropic glutamate receptor 2
mGlu5/mGluR5	Metabotropic glutamate receptor 5
MK-801	Dizocilpine
MMTS	Methyl methanethiosulfonate
NBT	Nitro blue tetrazolium
NMDA	N-methyl-D-aspartate
NMMA	N^G^-Monomethyl-L-arginine
nNOS	Neuronal nitric oxide synthase
NO	Nitric oxide
NP-40	Nonidet P-40
NPA	Nω-Propyl-L-arginine
PAM	Positive allosteric modulator
PMA	Phorbol 12-myristate 13-acetate
PMSF	Phenylmethylsulfonyl fluoride
ROS	Reactive oxygen species
SDMA	Symmetric dimethylarginine
SDS	Sodium dodecyl sulfate
SDS-PAGE	Sodium dodecyl sulfate–polyacrylamide gel electrophoresis
SNO	S-nitrosylated/S-nitrosylation-related
TBS-T	Tris-buffered saline with Tween 20
TCA	Trichloroacetic acid
VU0152100	M4 muscarinic receptor positive allosteric modulator
VU0357017	M1 muscarinic receptor positive allosteric modulator
