# Supplementary material for: Positive Allosteric Modulation of mGlu and Muscarinic Receptors Differentially Regulate Nitric Oxide-Dependent Signaling in a Scopolamine-Induced Model of Cognitive Impairment
Source: Med Sci (Basel). 2026 Oct 7;14(6):644. doi: 10.3390/medsci14060644 (PMC13706852; doi:10.3390/medsci14060644)
Supplement: Supplementary file 1 [file medsci-14-00644-s001.zip › medsci-4531505-supplementary.pdf]

Total-protein amido black staining- scopolamine

Blot A

M 1 2 3 4 5 6 7 8 9 10 11 12

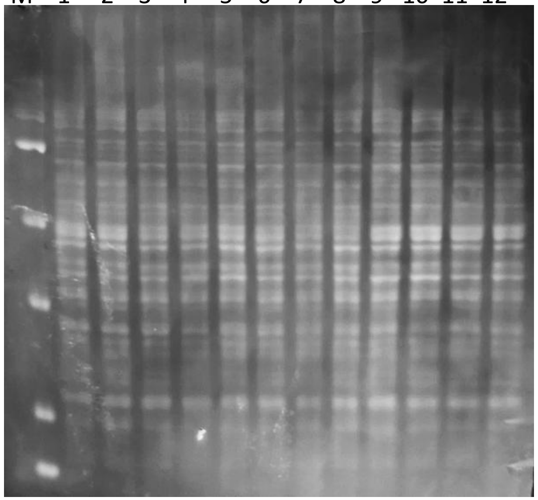

Blot B

M 1 2 3 4 5 6 7 8 9 10 11 12

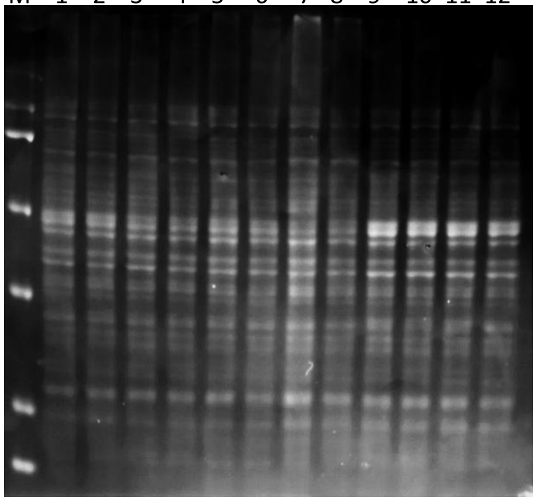

Blot C

M 1 2 3 4 5 6 7 8 9 10 11 12

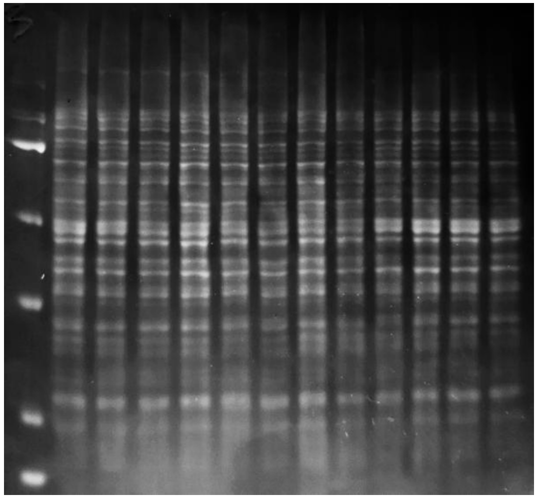

Blot D

M 1 2 3 4 5 6 7 8 9 10 11 12

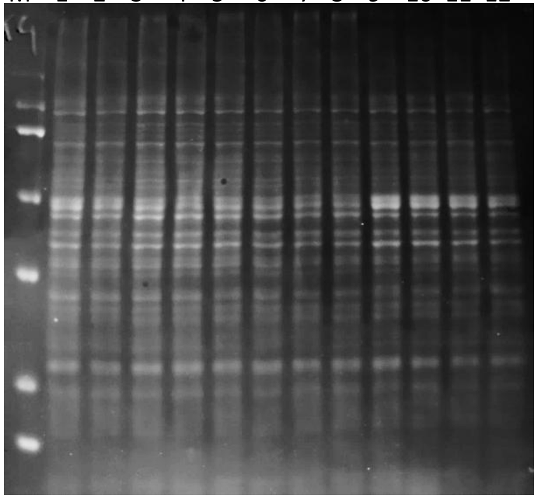

Blot E

M 1 2 3 4 5 6 7 8 9 10 11 12

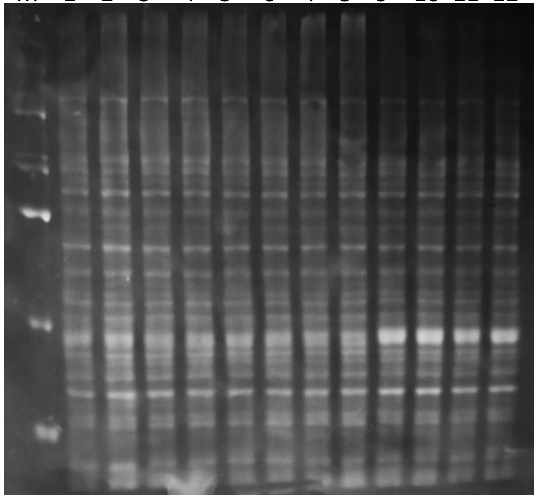

M-size marker
1,2-control
3,4- Scopolamine
5,6-Scop.+VU0152100
7,8-Scop.+VU0357017
9,10-Scop.+LY487379
11,12-Scop.+CDPPB

Total S-nitrosylation -scopolamine

Blot A

M 1 2 3 4 5 6 7 8 9 10 11 12

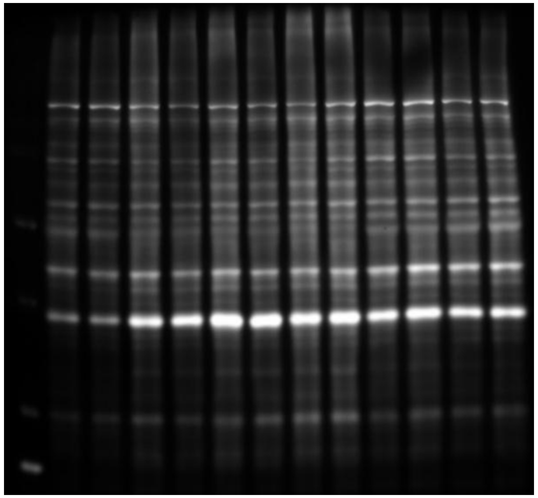

Blot B

M 1 2 3 4 5 6 7 8 9 10 11 12

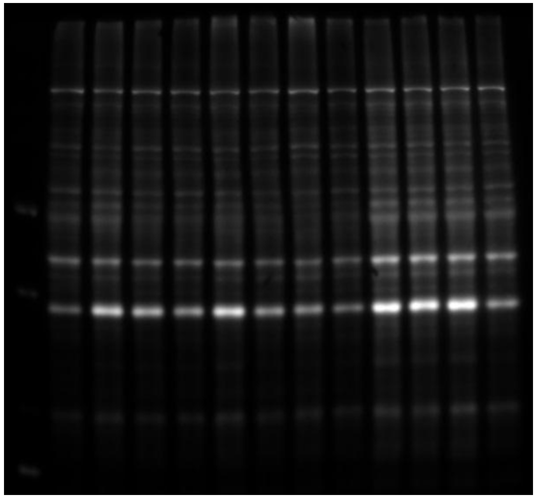

Blot C

M 1 2 3 4 5 6 7 8 9 10 11 12

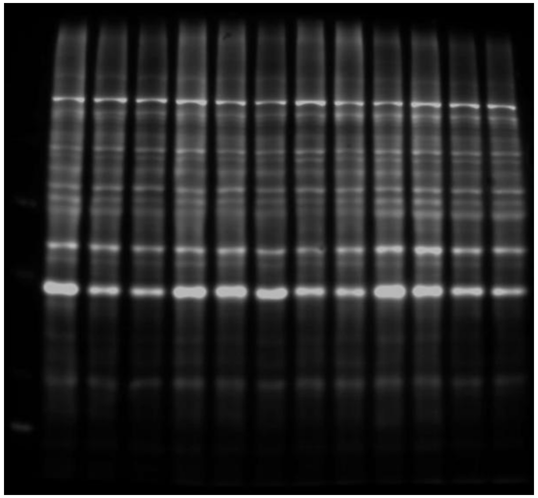

Blot D

M 1 2 3 4 5 6 7 8 9 10 11 12

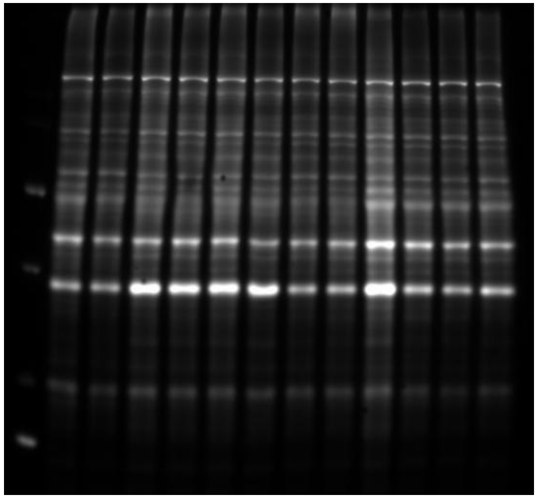

Blot E

M 1 2 3 4 5 6 7 8 9 10 11 12

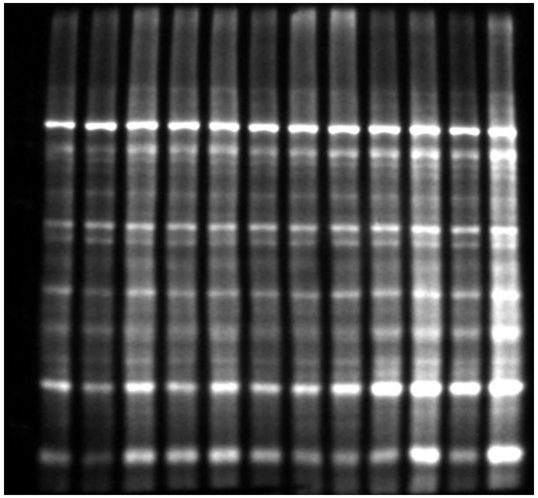

M-size marker
1,2-control
3,4- Scopolamine
5,6-Scop.+VU0152100
7,8-Scop.+VU0357017
9,10-Scop.+LY487379
11,12-Scop.+CDPPB

Total protein Actin (42kDa) and APP (103kDa)

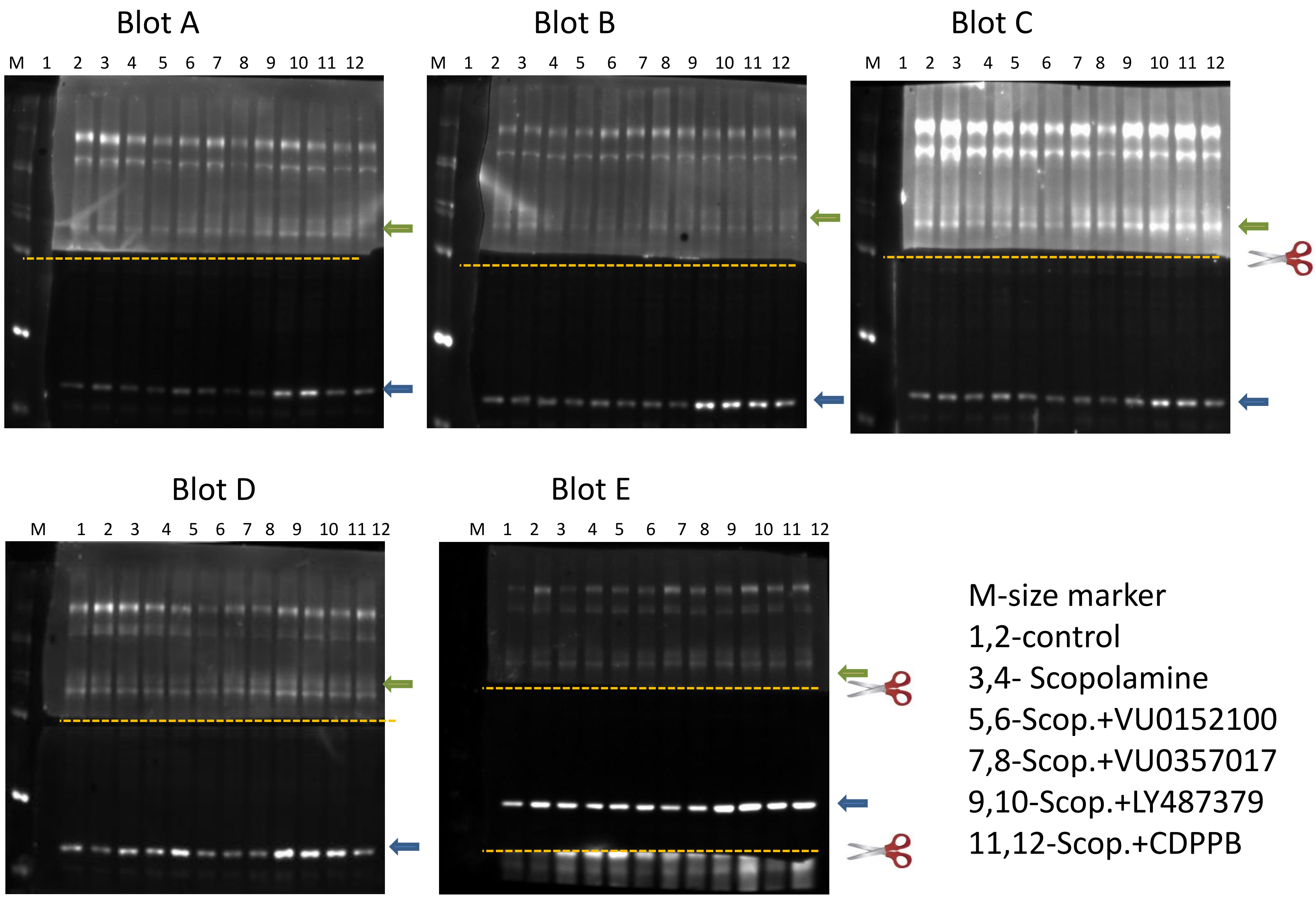

Total protrin Glt-1 (62kDa)

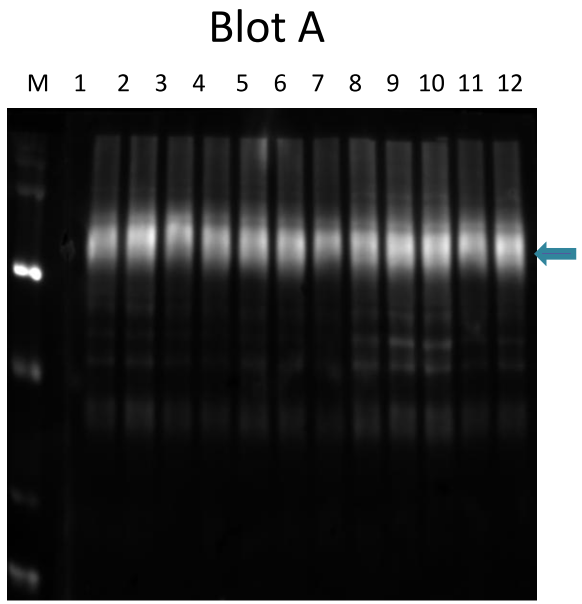

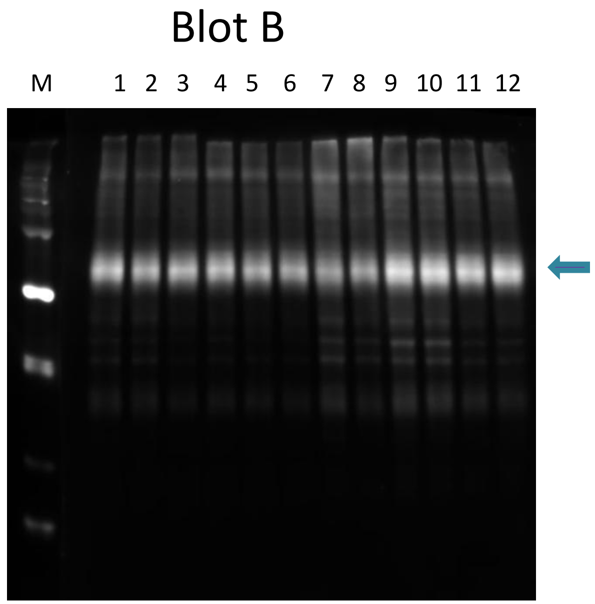

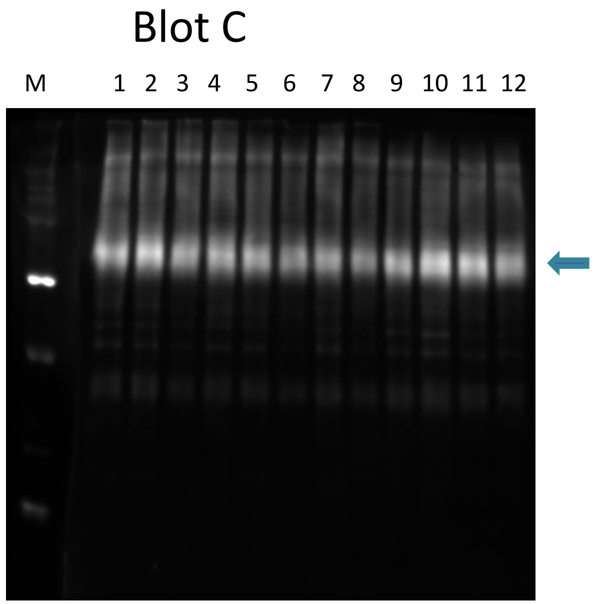

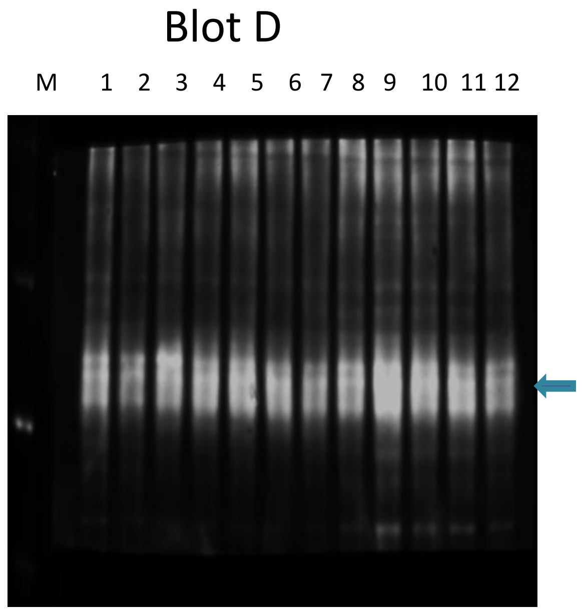

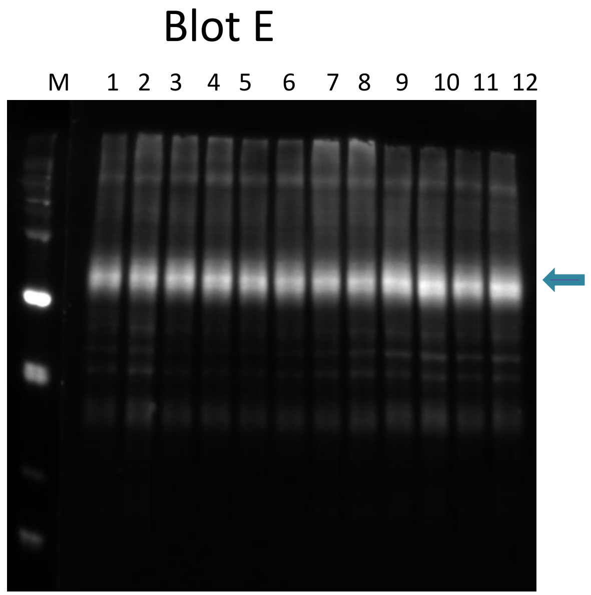

M-size marker
1,2-control
3,4- Scopolamine
5,6-Scop.+VU0152100
7,8-Scop.+VU0357017
9,10-Scop.+LY487379
11,12-Scop.+CDPPB

SNO-GLT-1 (62kDa)

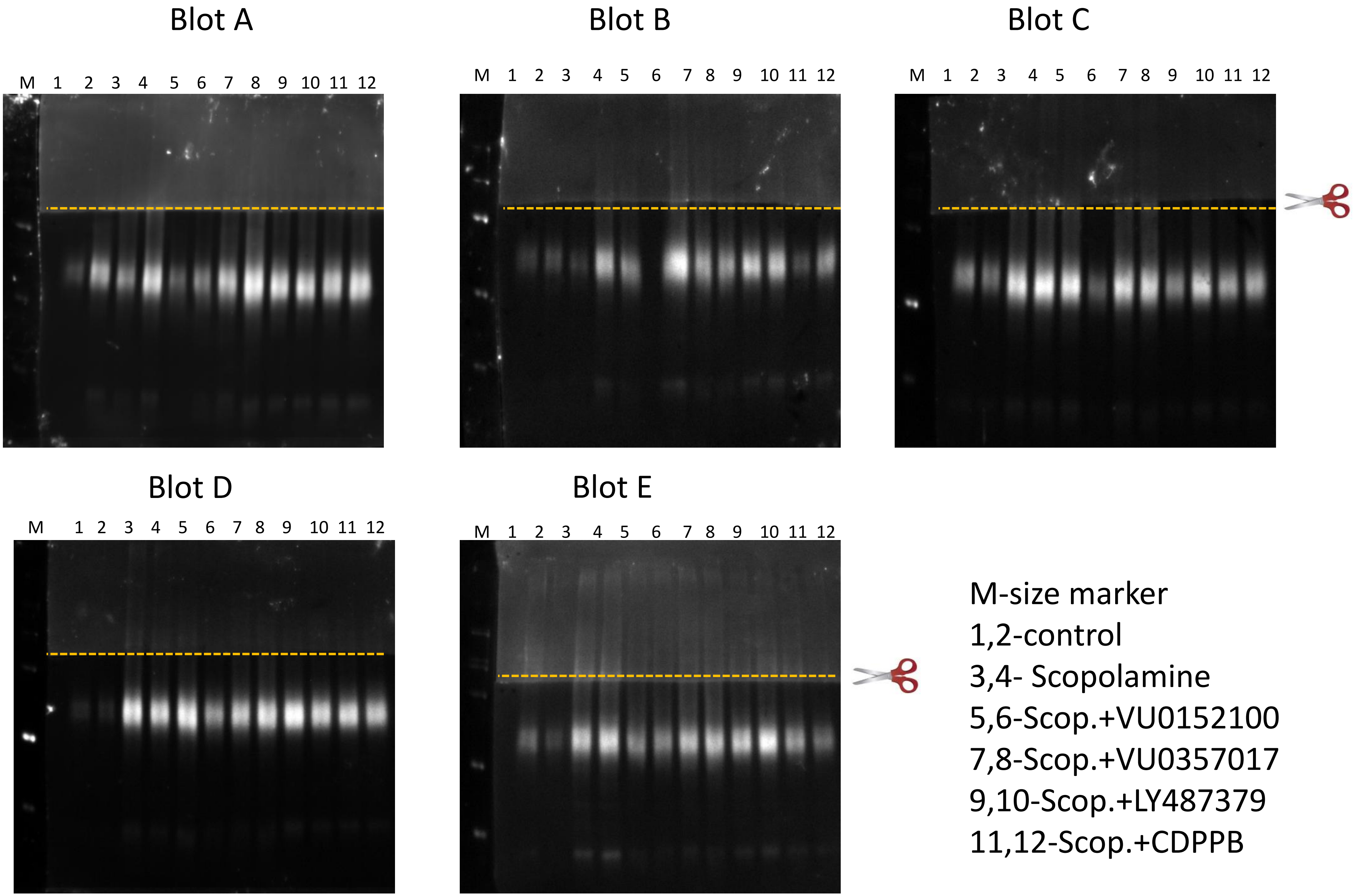

M-size marker
1,2-control
3,4- Scopolamine
5,6-Scop.+VU0152100
7,8-Scop.+VU0357017
9,10-Scop.+LY487379
11,12-Scop.+CDPPB
